# Impact of metabolic syndrome on postoperative outcomes of transsphenoidal pituitary surgery: analysis of U.S. nationwide inpatient sample data 2005–2018

**DOI:** 10.3389/fendo.2024.1235441

**Published:** 2024-03-25

**Authors:** Jiun-Lin Yan, Wan-Chin Kan, Yi-Hsien Kuo, Mao-Yu Chen, Pin-Yuan Chen, Kuan-Hao Fu

**Affiliations:** ^1^ Department of Neurosurgery, Keelung Chang Gung Memorial Hospital, Keelung, Taiwan; ^2^ School of Traditional Chinese Medicine, College of Medicine, Chang Gung University, Taoyuan, Taiwan; ^3^ Department of Radiology, Chang Gung Memorial Hospital, Linkou, Taiwan; ^4^ College of Medicine, Chang Gung University, Taoyuan, Taiwan

**Keywords:** metabolic syndrome (MetS), transsphenoidal pituitary surgery, pituitary adenoma, in-hospital outcomes, nationwide inpatient sample (NIS)

## Abstract

**Introduction:**

Transsphenoidal surgery (TSS) is the preferred surgical method for most pituitary adenomas owing to high efficacy and low mortality. This study aimed to evaluate the influence of metabolic syndrome (MetS) on postoperative outcomes of TSS for pituitary adenoma.

**Methods:**

This population-based, retrospective observational study extracted data of adults 20-79 y receiving TSS for pituitary adenoma from the US Nationwide Inpatient Sample (NIS) between 2005-2018. Primary outcomes were pituitary-related complications, poor outcomes (i.e., in-hospital mortality or unfavorable discharge), prolonged length of stay (LOS), and patient safety indicators (PSIs). Univariate and multivariate regressions were performed to determine the associations between study variables and outcomes.

**Results:**

19,076 patients (representing a 93,185 US in-patient population) were included, among which 2,109 (11.1%) patients had MetS. After adjustment, pre-existing MetS was not significantly associated with presence of pituitary-related complications and poor outcomes. In contrast, MetS was significantly associated with an increased risk for prolonged LOS (adjusted OR (aOR) = 1.19; 95% CI: 1.05-1.34), PSIs (aOR = 1.31; 95% CI: 1.07-1.59) and greater hospital costs (adjusted β = 8.63 thousand USD; 95% CI: 4.98-12.29). Among pituitary-related complications, MetS was independently associated with increased risk of cerebrospinal fluid (CSF) rhinorrhea (aOR = 1.22, 95% CI: 1.01, 1.47) but lowered diabetes insipidus (aOR = 0.83, 95% CI: 0.71, 0.97).

**Discussion:**

MetS does not pose excessive risk of in-hospital mortality or unfavorable discharge. However, MetS independently predicted having PSIs, prolonged LOS, greater hospital costs, and CSF rhinorrhea. Study findings may help clinicians achieve better risk stratification before TSS.

## Introduction

Pituitary adenomas, a common type of benign neuroendocrine tumor, originate from the adenohypophysis cells and account for approximately 10%-20% of all primary intracranial tumors, presenting a significant clinical challenge (1). It has been recently renamed as pituitary neuroendocrine tumor (PitNET) because invasive pituitary adenomas behave somewhat similarly to NETs, and pituitary hormone-producing cells are neuroendocrine cells (2). Surgical resection is the first-line treatment for most giant pituitary adenomas and can be performed using the transcranial approach or the transsphenoidal surgery (TSS) (3, 4). The TSS has gradually replaced craniotomy due to its lower impact on normal brain tissue and fewer postoperative complications (5).

Metabolic syndrome (MetS) is a group of metabolic-related disorders including abdominal obesity, insulin resistance, dyslipidemia, and hypertension (6). In the general population, MetS is associated with an increased risk of adverse health outcomes and perioperative complications (7). Pituitary adenoma may cause abnormal hormone secretion, which may have wide-ranging consequences, affecting metabolism throughout the body (8). Prior research suggests that metabolic dysregulation in pituitary adenoma patients correlates with a higher incidence of MetS and its markers than in the general population (9, 10).

Despite the prevalence of both MetS and pituitary adenomas, limited data exists on how pre-existing MetS impacts TSS outcomes. This study addresses this gap by evaluating the influence of MetS on TSS outcomes for patients with pituitary adenoma using a large dataset. The findings may help to enhance risk assessment and decision-making by shedding light on the role of MetS in TSS, contributing to more tailored patient management strategies.

## Methods

### Study design and data source

This population-based, retrospective observational study extracted all data from the US Nationwide Inpatient Sample (NIS) database, which is the largest all-payer, continuous inpatient care database in the United States, including about 8 million hospital stays each year (11). The database is administered by the Healthcare Cost and Utilization Project (HCUP) of the US National Institutes of Health (NIH) (https://hcup-us.ahrq.gov/db/nation/nis/nisdbdocumentation.jsp). Patient data include primary and secondary diagnoses, primary and secondary procedures, admission and discharge status, patient demographics, expected payment source, duration of hospital stay, and hospital characteristics (i.e., bed size/location/teaching status/hospital region). All admitted patients are initially considered for inclusion. The continuous, annually updated NIS database derives patient data from about 1,050 hospitals from 44 States in the US, representing a 20% stratified sample of US community hospitals as defined by the American Hospital Association (12).

### Ethics statement

All data were obtained through request to the Online HCUP Central Distributor. This study conforms to the NIS data-use agreement. Because this study analyzed pre-collected, de-identified data from the NIS and the public were not involved directly, our hospital exempted further Institutional Review Board (IRB) approval after the study protocol was submitted.

### Study population

The data of adults aged 20 to 79 years who had been diagnosed with pituitary adenoma and had undergone transsphenoidal pituitary surgery between 2005 and 2018 were extracted from the NIS database. Diagnoses and surgeries were confirmed using the International Classification of Diseases, Ninth Revision (ICD-9) and Tenth (ICD-10) diagnostic codes for pituitary adenoma (ICD-9: 227.3; ICD-10: D35.2) and transsphenoidal pituitary surgery (ICD-9-PCS: 07.62, 07.65; ICD-10-PCS: 0GB03, 0GB04, or 0GT04). Patients lacking complete data of in-hospital mortality, discharge destination; hospital costs, sex, household income, primary payer, admission type and hospital bed size were excluded.

### Study variables and outcome measures

The main exposure variable of MetS was defined according to the diagnostic codes for MetS (ICD-9: 277.7; ICD-10: E88.81) or having at least three of the five components of MetS, including BMI **≥** 30 kg/m^2^ (ICD-9: V85.3-V85.4; ICD-10: E66.0-E66.2, E66.8, E66.9, Z68.3-Z68.4), blood pressure ≥ 130/85 mmHg (ICD-9 codes: 401-405; ICD-10: I10-I16), fasting glucose ≥ 100 mg/dL (5.6 mmol/L) (ICD-9 codes: 250.x0, 250.x2; ICD-10: E11), serum HDL-C < 40 mg/dL in men and < 50 mg/dL in women (ICD-9: 272.5-272.6; ICD-10: E78.6, E88.1) and serum triglycerides ≥ 150 mg/dL (ICD-9 codes: 272.1-272.4; ICD-10: E78.1-E78.5). The definition we utilized is derived from the International Diabetes Federation (13).

Study outcomes were incidence of: 1) poor outcomes, a composite outcome defined by in-hospital mortality or unfavorable discharge (i.e., transfer to long-term care facilities); 2) pituitary-related complications defined by ICD diagnostic codes, including pituitary apoplexy, panhypopituitarism, corticoadrenal insufficiency, acquired hydrocephalus, meningitis, cerebrospinal fluid (CSF) rhinorrhea, diabetes insipidus, hypernatremia, and syndrome of inappropriate antidiuretic hormone secretion (SIADH); 3) presence of any patient safety indicators (PSIs); 4) prolonged length of stay (LOS), defined as LOS >= 75^th^ percentile; and 5) hospital costs. Specifically, the PSIs used in the present study were developed by the Agency for Healthcare Research and Quality’s (AHRQ) of the US to simplify the common inpatient complications or adverse events that indicate compromised patient safety. The PSIs include as follow: anesthetic complications – misplaced endotracheal tube, adverse effects in therapeutic use, other CNS depressants and anesthetics, poisoning by other CNS depressants and anesthetics, pressure ulcer, foreign body retained after procedure/surgery, iatrogenic pneumothorax, central venous line infection, postoperative hip fracture, postoperative physiologic & metabolic derangement – secondary diabetes with ketoacidosis, postoperative hemorrhage, postoperative respiratory failure, deep vein thrombosis & pulmonary embolism, sepsis, postoperative wound dehiscence, accidental puncture or laceration and transfusion reaction. The details and validation of the PSI in pituitary tumor patients was previously described elsewhere (14, 15).

### Covariates

Patients’ baseline characteristics included age, sex, household income, insurance status (primary payer), smoking status, and severity of comorbidities assessed by Charlson comorbidity index (CCI). Weekend admission, emergent admission and hospital-related characteristics (bed number, location/teaching status, hospital region) were also extracted from the database as part of the comprehensive data available for all patients.

### Statistical analysis

Weighted samples (TRENDWT and DISCWT used before 2011 and after 2012, respectively), stratum (NIS_STRATUM), and cluster (HOSPID) were used to estimate the nationwide analyses. Descriptive statistics are expressed as unweighted counts (n) and weighted percentages (%) for categorical data with the SAS procedure of PROC SURVEYFREQ and mean ± standard error (SE) with PROC SURVEYMEANS for continuous data. Comparison between groups were conducted using the same SAS procedures. Logistic regressions were used to calculate the odds ratios (ORs) and 95% confidence intervals (CIs) to determine associations between MetS and outcomes using SAS procedure of PROC SURVEYLOGISTIC. Linear regressions were performed to analyze beta estimates and 95% CI of each factor associated with total hospital costs using PROC SURVEYREG. Multivariate models were adjusted for variables that were significant (p < 0.05) in univariate analyses. Stratified analysis to identify the associations between pituitary-related complication and MetS according to type of pituitary adenoma (secreting or non-secreting) was also performed. The statistical software package SAS software version 9.4 (SAS Institute Inc., Cary, NC, USA) was used for all statistical analyses.

## Results

**Figure 1:**
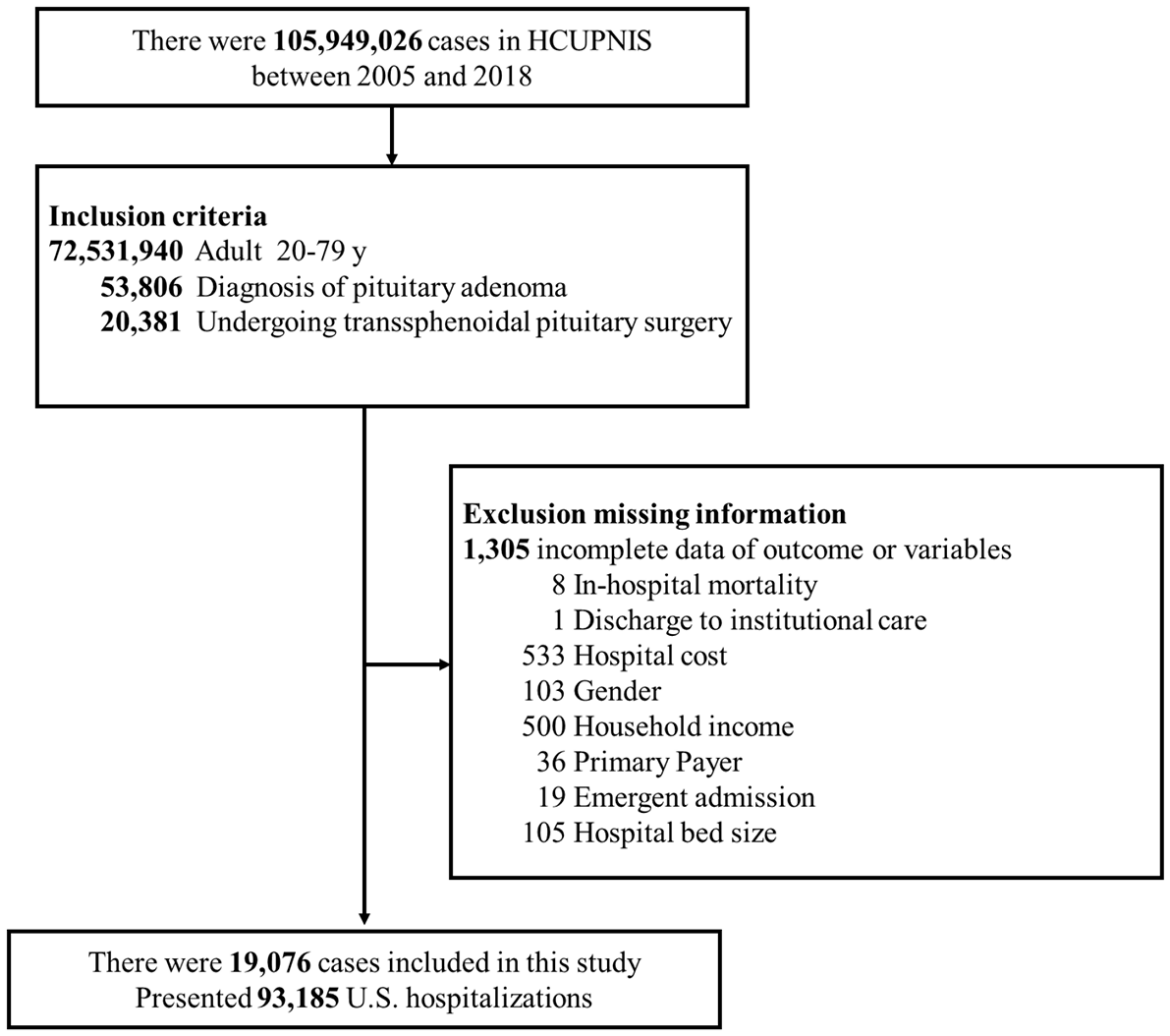
Flow diagram of patient selection.

The patient selection process is depicted in **Figure 1**. Initially, 20,381 patients aged from 20 to 79 years with pituitary adenoma and undergoing TSS during 2005-2018 were identified in the NIS database. Patients with incomplete data of outcomes and main variables of interest were excluded (n = 1,305). Finally, 19,076 patients (representing a 93,185 US in-patient population) were included as the study cohort, among which 2,109 (11.1%) patients were diagnosed with MetS (**Figure 1**).

### Characteristics of patients undergoing TSS for pituitary adenoma

Patients’ characteristics and outcomes following TSS for pituitary adenoma are summarized in **Table 1**. Mean age of the study cohort was 52.1 ± 0.1 years, while 9,649 (50.6%) patients were females and 10,012 (52.6%) patients were White. Mean total hospital costs was 69.8 ± 1.2 thousand USD. Patients with MetS were older, and a greater percentage were female, Black, with lower household income, and insurance was covered by Medicare/Medicaid as compared to those without MetS. Higher percentages of smoking (26.1% vs. 20.5%, p < 0.001) and greater Charlson comorbidity index (CCI) (1: 20.8 vs. 14.3%; 2+: 14.8% vs. 6.1%, p < 0.001) were observed in patients with MetS. Non-secreting tumors were fewer in patients with MetS than in those without MetS (76.8% vs. 83.2%, p < 0.001). Higher frequencies of emergent admission (17.9% vs. 13.8%, p < 0.001), weekend admission (4.4% vs. 3.4%, p = 0.032), and staying in mid-Western or Southern hospitals rather than other regions (Midwest: 24.8% vs. 18.4%; South: 38.8% vs. 36.1%, p < 0.001) were also observed in patients with MetS. Distribution of the components of MetS in the entire study cohort is summarized in **Supplementary Table S1**.

**Table 1:** Characteristics of study population with and without MetS.

	Total (n=19,076)	MetS		P-value
		Yes (n=2,109)	No (n=16,967)	
Demography				
Age, years	52.1 ± 0.1	57.5 ± 0.3	51.5 ± 0.1	**<0.001**
20-29	1622 (8.5)	39 (1.8)	1583 (9.3)	**<0.001**
30-39	2634 (13.8)	146 (6.9)	2488 (14.6)	
40-49	3643 (19.1)	350 (16.6)	3293 (19.4)	
50-59	4554 (23.9)	560 (26.5)	3994 (23.5)	
60-69	4087 (21.5)	645 (30.5)	3442 (20.3)	
70-79	2536 (13.3)	369 (17.6)	2167 (12.8)	
Sex				**0.003**
Male	9427 (49.4)	978 (46.2)	8449 (49.8)	
Female	9649 (50.6)	1131 (53.8)	8518 (50.2)	
Race				**<0.001**
White	10012 (52.6)	1032 (49.0)	8980 (53.0)	
Black	2942 (15.5)	473 (22.5)	2469 (14.6)	
Hispanic	2169 (11.4)	232 (11.1)	1937 (11.5)	
Others	1466 (7.7)	127 (6.1)	1339 (7.9)	
Unknown	2487 (12.8)	245 (11.4)	2242 (12.9)	
Household income				**<0.001**
Quartile1	4542 (23.8)	614 (29.2)	3928 (23.1)	
Quartile2	4551 (23.8)	534 (25.3)	4017 (23.6)	
Quartile3	4806 (25.2)	507 (24.1)	4299 (25.4)	
Quartile4	5177 (27.2)	454 (21.5)	4723 (27.9)	
Primary Payer				**<0.001**
Medicare/Medicaid	6400 (33.6)	985 (46.8)	5415 (32.0)	
Private including HMO	11068 (58.0)	985 (46.6)	10083 (59.4)	
Self-pay/no-charge/other	1608 (8.4)	139 (6.6)	1469 (8.6)	
Smoking				**<0.001**
No	15051 (78.8)	1561 (73.9)	13490 (79.5)	
Yes	4025 (21.2)	548 (26.1)	3477 (20.5)	
CCI				**<0.001**
0	14880 (77.9)	1358 (64.5)	13522 (79.6)	
1	2859 (15.0)	440 (20.8)	2419 (14.3)	
2+	1337 (7.0)	311 (14.8)	1026 (6.1)	
Type of pituitary adenoma				**<0.001^c^**
Non-secreting	15736 (82.5)	1620 (76.8)	14116 (83.2)	
Secreting				
Acromegaly	1417 (7.4)	123 (5.8)	1294 (7.6)	
Prolactinoma	402 (2.1)	28 (1.4)	374 (2.2)	
TSHoma	10 (0.1)	–	10 (0.1)	
Cushing’s	1511 (7.9)	338 (16.0)	1173 (6.9)	
Hospital characteristics				
Emergent admission				**<0.001**
No	16361 (85.7)	1732 (82.1)	14629 (86.2)	
Yes	2715 (14.3)	377 (17.9)	2338 (13.8)	
Weekend admission				**0.032**
No	18408 (96.5)	2016 (95.6)	16392 (96.6)	
Yes	668 (3.5)	93 (4.4)	575 (3.4)	
Hospital bed size				0.872
Small	1126 (5.9)	119 (5.6)	1007 (5.9)	
Medium	2590 (13.6)	289 (13.7)	2301 (13.6)	
Large	15360 (80.5)	1701 (80.7)	13659 (80.5)	
Hospital location/teaching status				0.062
Rural	236 (1.2)	32 (1.4)	204 (1.2)	
Urban nonteaching	2291 (11.9)	226 (10.7)	2065 (12.1)	
Urban teaching	16549 (86.9)	1851 (87.9)	14698 (86.7)	
Hospital region				**<0.001**
Northeast	3685 (19.4)	356 (17.0)	3329 (19.7)	
Midwest	3630 (19.1)	524 (24.8)	3106 (18.4)	
South	6986 (36.4)	821 (38.8)	6165 (36.1)	
West	4775 (25.0)	408 (19.4)	4367 (25.7)	

Categorical variables are presented as unweighted counts (weighted percentage) and continuous variables are presented as mean ± SE.

HMO, Health Maintenance Organization; CCI, Charlson comorbidity index; TSHoma, thyrotropinoma, MetS, metabolic syndrome. P-value < 0.05 are showed in bold.

### Outcomes after TSS

Postoperative outcomes of TSS are documented in **Table 2**, including: 2.1% patients had pituitary-related complications, 3.1% had poor outcomes after surgery, 19.2% had prolonged LOS, and 4.2% had the presence of any PSI. Patients with MetS also had an overall greater proportion of poor outcomes (5.6% vs. 2.8%, p < 0.001), prolonged LOS (25.5% vs 18.4%, p < 0.001), and any PSI (6.7% vs 3.9%, p < 0.001). More specifically, among pituitary-related complications, patients with MetS had significantly more CSF rhinorrhea (6.5% vs 5.3%, p < 0.027) and hypernatremia (6.5% vs. 5.3%, p < 0.001).

**Table 2:** In-hospital outcomes of study population with and without MetS.

	Total (n=19,076)	MetS		P-value
		Yes (n=2,109)	No (n=16,967)	
**Pituitary-related complications**	6107 (32.1)	696 (33.1)	5411 (32.0)	0.336
Pituitary apoplexy	457 (2.4)	59 (2.8)	398 (2.3)	0.225
Panhypopituitarism	1560 (8.2)	182 (8.7)	1378 (8.2)	0.466
Corticoadrenal insufficiency	1364 (7.2)	167 (8.0)	1197 (7.1)	0.145
Acquired hydrocephalus	169 (0.9)	19 (0.9)	150 (0.9)	0.972
Meningitis	39 (0.2)	2 (0.1)	37 (0.2)	0.181
CSF rhinorrhea	1029 (5.5)	136 (6.5)	893 (5.3)	**0.027**
Diabetes insipidus	2371 (12.4)	206 (9.8)	2165 (12.8)	**<0.001**
Hypernatremia	1037 (5.5)	151 (7.1)	886 (5.3)	**<0.001**
SIADH	459 (2.4)	43 (2.0)	416 (2.5)	0.226
**Poor outcomes**	591 (3.1)	120 (5.6)	471 (2.8)	**<0.001**
**Prolonged LOS ^a,b^**	3655 (19.2)	538 (25.5)	3117 (18.4)	**<0.001**
**PSI**	805 (4.2)	141 (6.7)	664 (3.9)	**<0.001**
Anesthetic complications – misplaced endotracheal tube	–	–	–	–
Anesthetic complications – adverse effects in therapeutic use, other CNS depressants and anesthetics	1 (0.0)	1 (0.0)	–	–
Anesthetic complications – poisoning by other CNS depressants and anesthetics	4 (0.0)	1 (0.0)	3 (0.0)	**0.031**
Pressure ulcer	19 (0.1)	2 (0.1)	17 (0.1)	0.921
Foreign body retained after procedure/surgery	1 (0.0)	1 (0.0)	–	–
Iatrogenic pneumothorax	2 (0.0)	–	2 (0.0)	–
Central venous line infection	8 (0.0)	–	8 (0.0)	–
Postoperative hip fracture	1 (0.0)	–	1 (0.0)	–
Postoperative physiologic & metabolic derangement – secondary diabetes with ketoacidosis	38 (0.2)	21 (1.0)	17 (0.1)	**<0.001**
Postoperative hemorrhage	219 (1.1)	25 (1.2)	194 (1.1)	0.829
Postoperative respiratory failure	237 (1.3)	47 (2.2)	190 (1.1)	**<0.001**
Deep vein thrombosis & pulmonary embolism	179 (0.9)	37 (1.8)	142 (0.8)	**<0.001**
Sepsis	92 (0.5)	17 (0.8)	75 (0.4)	**0.022**
Postoperative wound dehiscence	9 (0.0)	1 (0.0)	8 (0.0)	0.987
Accidental puncture or laceration	150 (0.8)	11 (0.5)	139 (0.8)	0.158
Transfusion reaction	3 (0.0)		3 (0.0)	
**Total hospital costs**	69.8 ± 1.2	83.4 ± 1.7	68.1 ± 1.2	**<0.001**

Categorical variables are presented as unweighted counts (weighted percentage) and continuous variables are presented as mean ± SE.

CSF, cerebrospinal fluid; LOS, length of stay; SIADH, syndrome of inappropriate antidiuretic hormone secretion; CNS, central nervous system; HMO, Health Maintenance Organization; CCI, Charlson comorbidity index; MetS, metabolic syndrome; PSI, patient safety indicators; TSHoma, thyrotropinoma.

a Excluded in-hospital mortality patients.

b LOS >75^th^ percentile (5 days).

c Since Rao-Scott chi-square tests cannot be computed for the table by group based on Secreting because at least one table cell has 0 frequency, the Fisher’s exact test was used. P-value < 0.05 are showed in bold.

### Associations between MetS and pituitary-related complications, poor outcomes, prolonged LOS, PSIs, and total hospital costs

Associations between MetS and pituitary-related complications, poor outcomes, prolonged LOS, PSI, and total hospital costs are summarized in **Table 3**. After adjusting for all relevant confounders from univariate analysis in the multivariable models, MetS was not significantly associated with the presence of pituitary-related complications and poor outcomes. Although MetS was significantly associated with a slightly increased risk for prolonged LOS (adjusted OR (aOR) = 1.19; 95% CI: 1.05-1.34). In addition, subjects with MetS were more prone to have PSIs (aOR = 1.31; 95% CI: 1.07-1.59) and higher hospital costs (adjusted β = 8.63 thousand USD; 95% CI: 4.98- 12.29). The complete models of the associations are shown in **Supplementary Tables S2**, **S3**.

**Table 3:** Associations between MetS and outcomes.

Outcomes	Metabolic Syndrome (n=2,109 vs. 16,967)	Univariate	Multivariate
		Crude OR (95% CI)/β (95% CI)	Adjusted OR (95% CI)/β (95% CI)
**Pituitary-related complications** ^a^	Yes vs. No	1.05 (0.95, 1.16)	0.97 (0.87, 1.08)
**Poor outcomes** ^b^	Yes vs. No	**2.09 (1.71, 2.56)**	1.17 (0.93, 1.46)
In-hospital mortality ^c^	Yes vs. No	0.95 (0.44, 2.07)	0.50 (0.23, 1.12)
Unfavorable discharge ^d,h^	Yes vs. No	**2.25 (1.82, 2.77)**	1.25 (0.997, 1.58)
**Prolonged LOS ^e,h,i^**	Yes vs. No	**1.51 (1.36, 1.68)**	**1.19 (1.05, 1.34)**
PSI ^f^	Yes vs. No	**1.77 (1.46, 2.14)**	**1.31 (1.07, 1.59)**
**Total hospital cost (per 1000 dollars) ^g,j^**	Yes vs. No	**15.29 (11.62, 18.96)**	**8.63 (4.98, 12.29)**

Variables with p<0.05 in univariate analysis were adjusted for multivariate analysis.

Significant values are shown in bold.

In multivariate analysis, models were adjusted using the following covariates:

a sex, race, primary payer, categorical CCI, type of pituitary adenoma, emergent admission, weekend admission, hospital bed size, and hospital location/teaching status.

b age, sex, race, household income, primary payer, smoking, categorical CCI, type of pituitary adenoma, emergent admission, weekend admission, hospital bed size, and hospital region.

c age, sex, race, primary payer, categorical CCI, type of pituitary adenoma, emergent admission, weekend admission, hospital bed size, and hospital location/teaching status.

d age, sex, race, household income, primary payer, categorical CCI, type of pituitary adenoma, emergent admission, weekend admission, hospital bed size, and hospital region.

e age, race, household income, primary payer, categorical CCI, type of pituitary adenoma, emergent admission, weekend admission, hospital bed size, and hospital region.

f age, sex, primary payer, categorical CCI, emergent admission, and weekend admission.

g age, sex, race, primary payer, categorical CCI, type of pituitary adenoma, emergent admission, weekend admission, hospital bed size, hospital location/teaching status, and hospital region.

h Excluded in-hospital mortality patients.

i LOS >75^th^ percentile (5 days).

j Adjusted β was used to total hospital cost (per 1000 dollars).

LOS, length of stay; CCI, Charlson comorbidity index; OR, odds ratio; CI, confidence interval; MetS, metabolic syndrome; PSI, patient safety indicator.

Sensitivity analyses conducted on patients who met at least two of five criteria for MetS revealed similar findings, as shown in **Supplementary Table S4**.

### Associations between MetS and specific pituitary-related complications

**Figure 2:**
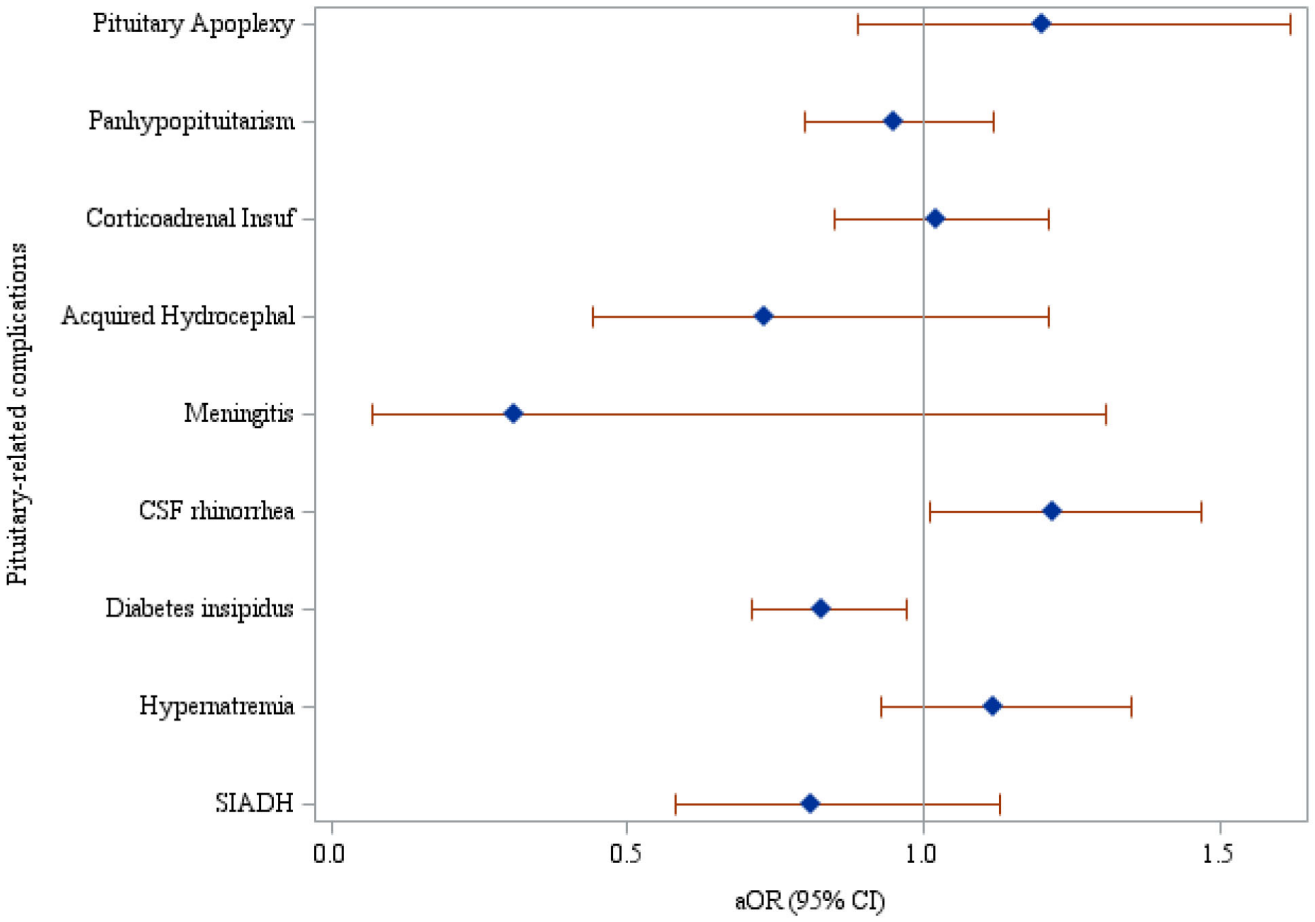
Forest plot of the associations between MetS and each pituitary-related complication.

Associations between MetS and the specific pituitary-related complications, including pituitary apoplexy, panhypopituitarism, corticoadrenal insufficiency, acquired hydrocephalus, meningitis, CSF rhinorrhea, diabetes insipidus, hypernatremia, and SIADH are documented in **Table 4** and **Figure 2**. After adjusting multivariate analysis for significant variables from univariate analysis, patients with MetS had a significantly higher risk of CSF rhinorrhea (aOR, 1.22; 95% CI: 1.01-1.47) and a lower risk of diabetes insipidus (aOR, 0.83; 95% CI: 0.71-0.97). Sensitivity analyses conducted on patients who met at least two of five criteria for MetS showed no significant association with CSF rhinorrhea. However, similar to the original results, there was a significantly reduced risk of developing SIADH, with an aOR of 0.77 (95% CI: 0.61-0.96), as detailed in **Supplementary Table S5**.

**Table 4:** Associations between MetS and specific pituitary-related complications.

Pituitary-related complication	MetS (n=2,109 vs. 16,967)	Univariate	Multivariate
		Crude OR (95% CI)	Adjusted OR (95% CI)
Pituitary apoplexy ^a^	Yes vs. No	1.19 (0.90, 1.57)	1.20 (0.89, 1.62)
Panhypopituitarism ^b^	Yes vs. No	1.06 (0.90, 1.25)	0.95 (0.80, 1.12)
Corticoadrenal insufficiency ^c^	Yes vs. No	1.13 (0.96, 1.34)	1.02 (0.85, 1.21)
Acquired hydrocephalus ^d^	Yes vs. No	0.99 (0.61, 1.60)	0.73 (0.44, 1.21)
Meningitis ^e^	Yes vs. No	0.40 (0.10, 1.61)	0.31 (0.07, 1.31)
CSF rhinorrhea ^f^	Yes vs. No	**1.23 (1.02, 1.49)**	**1.22 (1.01, 1.47)**
Diabetes insipidus ^g^	Yes vs. No	**0.74 (0.64, 0.86)**	**0.83 (0.71, 0.97)**
Hypernatremia ^h^	Yes vs. No	**1.38 (1.15, 1.66)**	1.12 (0.93, 1.35)
SIADH ^i^	Yes vs. No	0.82 (0.60, 1.13)	0.77 (0.55, 1.08)

Variables with p<0.05 in univariate analysis were adjusted for multivariate analysis.

Significant values are shown in bold.

In multivariate analysis, models were adjusted with the following covariates:

a age, race, categorical CCI, type of pituitary adenoma, emergent admission, and weekend admission.

b age, sex, primary payer, smoking, race, categorical CCI, type of pituitary adenoma, emergent admission, weekend admission, hospital bed size, hospital location/teaching status, and hospital region.

c sex, primary payer, smoking, categorical CCI, type of pituitary adenoma, emergent admission, weekend admission, hospital bed size, hospital location/teaching status, and hospital region.

d age, sex, primary payer, categorical CCI, type of pituitary adenoma, emergent admission, and weekend admission.

e categorical CCI, type of pituitary adenoma, emergent admission, weekend admission, and hospital location/teaching status.

f sex, race, emergent admission, hospital bed size, and hospital location/teaching status.

g age, sex, primary payer, categorical CCI, type of pituitary adenoma, emergent admission, weekend admission, and hospital location/teaching status.

h age, sex, primary payer, categorical CCI, type of pituitary adenoma, emergent admission, and weekend admission.

i age, sex, race, household income, categorical CCI, type of pituitary adenoma, emergent admission, weekend admission, and hospital location/teaching status.

CSF, cerebrospinal fluid; SIADH, syndrome of inappropriate antidiuretic hormone secretion; CCI, Charlson comorbidity index; OR, odds ratio; CI, confidence interval; MetS, metabolic syndrome.

### Associations between MetS and postoperative outcomes stratified by type of pituitary tumor (secreting and non-secreting)

We further carried out stratified analysis according to type of pituitary adenomas (secreting and non-secreting). The results are shown in **Table 5**. In patients with non-secreting pituitary adenoma, those with MetS exhibit a notably increased risk of prolonged LOS (aOR, 1.15; 95% CI: 1.01-1.32) and PSI (aOR, 1.33; 95% CI: 1.07-1.65). Meanwhile, patients with secreting pituitary adenoma and MetS display a significantly elevated risk of poor outcome (aOR, 2.04; 95% CI: 1.22-3.42) and prolonged LOS (aOR, 1.49; 95% CI: 1.17-1.90).

**Table 5:** Associations between MetS and outcomes stratified by the secreting pituitary adenoma or not.

Stratified variable/Outcomes	MetS (n=2,109 vs. 16,967)	Multivariate	
		Adjusted OR (95% CI)	p-value
Non-secreting			
Pituitary-related complications ^a^	Yes vs. No	0.98 (0.87, 1.10)	0.719
Poor outcomes ^b^	Yes vs. No	1.10 (0.86, 1.42)	0.436
In-hospital mortality c	Yes vs. No	0.56 (0.24, 1.33)	0.192
Unfavorable discharge ^d,g,^	Yes vs. No	1.17 (0.91, 1.52)	0.229
Prolonged LOS ^e,g,h^	Yes vs. No	**1.15 (1.01, 1.32)**	**0.039**
PSI ^f^	Yes vs. No	**1.33 (1.07, 1.65)**	**0.010**
Secreting			
Pituitary-related complications ^a^	Yes vs. No	0.98 (0.79, 1.22)	0.850
Poor outcomes ^b^	Yes vs. No	**2.04 (1.22, 3.42)**	**0.007**
In-hospital mortality ^c^	Yes vs. No	0.19 (0.01, 3.29)	0.256
Unfavorable discharge ^d,g^	Yes vs. No	**2.45 (1.42, 4.21)**	**0.001**
Prolonged LOS ^e,g,h^	Yes vs. No	**1.49 (1.17, 1.90)**	**0.001**
PSI ^f^	Yes vs. No	1.12 (0.71, 1.77)	0.637

Variables with p<0.05 in univariate analysis were adjusted for multivariate analysis (expect for the stratified variable). Significant values are shown in bold.

In multivariate analysis, models were adjusted with the following covariates:

a sex, race, primary payer, categorical CCI, emergent admission, weekend admission, hospital bed size, and hospital location/teaching status.

b age, sex, race, household income, primary payer, smoking, categorical CCI, emergent admission, weekend admission, hospital bed size, and hospital region.

c age, sex, race, primary payer, categorical CCI, emergent admission, weekend admission, hospital bed size, and hospital region.

d age, sex, race, household income, primary payer, categorical CCI, emergent admission, weekend admission, hospital bed size, and hospital region.

e age, race, household income, primary payer, categorical CCI, emergent admission, weekend admission, hospital bed size, and hospital region.

f age, sex, primary payer, categorical CCI, emergent admission, and weekend admission.

g Excluded in-hospital mortality patients.

h LOS >75th percentile (5 days).

LOS, length of stay; CCI, Charlson comorbidity index; OR, odds ratio; CI, confidence interval; MetS, metabolic syndrome; PSI, patient safety indicator.

## Discussion

To the best of our knowledge, this study is the first in the medical literature to evaluate the effects of MetS on short-term outcomes after TSS in patients with pituitary adenomas. Overall, MetS patients were older, with a higher proportion of women and Blacks. Patients with MetS had fewer non-secreting tumors than those without MetS. About one-third of patients with pituitary adenoma undergoing TSS had pituitary-related complications. Results of the present study have demonstrated that pre-existing MetS carries no undue risk of adverse outcomes, including in-hospital death or discharge to a long-term care facility. However, patients with MetS were more prone to have PSIs. MetS independently predicted longer hospital stays and higher hospital costs, as well as higher risk of CSF rhinorrhea.

Because no previous study has evaluated the role of MetS in outcomes of TSS for pituitary adenoma, direct comparison with previous studies cannot be done. However, two studies reported that high BMI, an indicator of overweight status, may lead to worse postoperative outcomes for TSS. Postoperative CSF leakage can also be a serious complication after transsphenoidal surgery, and elevated BMI is an important risk factor for spontaneous cerebrospinal fluid leak. A previous study identified elevated BMI as an independent predictor of CSF leakage after transsphenoidal transnasal endoscopic approach (16). That study recommended that patients with a BMI greater than 30 kg/m^2^ should undergo careful sellar reconstruction at the time of surgery and should receive close postoperative monitoring. Endoscopic pituitary surgery is increasingly used to treat pituitary lesions. Obesity, which is a growing epidemic worldwide and a MetS component, is associated with a number of comorbidities known to affect surgical outcomes. Another multi-institutional database study assessed the association between BMI and postoperative outcomes after endoscopic pituitary surgery (17). That study indicated that endoscopic pituitary surgery may be a safer treatment option for pituitary lesions in obese people. Although obese patients undergoing endoscopic pituitary surgery had an increased risk of medical complications and prolonged operative time, this did not affect mortality, reoperation, or readmission.

Our study found that MetS is an independent predictor of CSF rhinorrhea, more PSIs, longer stays and higher total costs. These findings may be elucidated through the following considerations. The structural and physiological challenges posed by MetS (e.g., obesity and hypertension) could contribute to an elevated risk of CSF rhinorrhea. For example, obesity (18) and chronic hypertension (19) are linked to a heightened risk of elevated intracranial pressure (ICP). This increased ICP can exert extra stress on the surgical site after TSS, potentially forcing CSF through any minor defects left in the sellar floor and causing a leak. Hyperglycemia impairs the body’s healing process by interfering with white blood cell function, essential for wound healing, and by inhibiting collagen formation, vital for tissue repair (20). Such compromised healing can further weaken the surgical site, elevating the risk of CSF leakage. Elevated triglycerides can induce systemic inflammation, undermining the body’s capacity to heal after surgery (21). Collectively, these alterations increase the body’s vulnerability to surgical stress, leading to enhanced postoperative inflammation and prolonged recovery periods. Furthermore, they necessitate specialized postoperative care, including meticulous blood sugar control and blood pressure management. Such comprehensive care requirements can prolong hospitalization and escalate the need for medical resources, consequently driving up hospital costs.

On the other hand, the discrepancy in MetS prevalence, particularly the lower prevalence in our studied population compared to broader estimates has been noted (22). This underestimation could arise from multiple factors. The data collected in the hospital setting might not fully capture the cases of MetS that exist in the population. There could be various reasons for under-coding, such as limited awareness among healthcare providers about MetS, or administrative issues in coding patient records. For example, the hospital’s coding system has constraints, allowing limited diagnoses per patient, resulting in prioritization of more severe clinical conditions over MetS in the coding process. We were aware of that the potential misclassification might hinder the results of the analyses, and therefore, it is advised to exercise caution when interpreting the results.

Furthermore, This study observed a perplexing trend where patients undergoing TSS with MetS displayed a decreased chance to diabetes insipidus, warranting further investigation for explanations. However, it’s imperative to recognize the possibility of undisclosed variables that could be impacting these findings. Notably, factors like tumor size and alterations in tumor cavity diameter, identified as potential contributors to diabetes insipidus risk in a previous study (23), might be exerting influence over the observed outcome. Regrettably, due to the absence of this specific data, we were constrained in our ability to conduct a more comprehensive analysis targeting these confounding factors.

### Limitations

First, the present study is inherently limited by its retrospective and observational nature, which limits the extent to which results can be generalized to other populations. Second, the possibility of coding errors exists as in other studies that used ICD code systems. Third, the intraoperative parameters such as duration of operation, blood loss, tumor size and biochemical data were not recorded by the NIS database thus could not be further investigated. Also, microscopic and endonasal endoscopic procedures could not be distinguished. Fourth, because data for each inpatient is confined to the time of a single hospitalization, this study also lacks follow-up data after discharge, precluding the analyses on the late complications after TSS. Fifth, conditions such as acromegaly and Cushing’s disease, which are linked to MetS components, could potentially be subject to underdiagnosis or divergent classification. Sixth, another important limitation of this study is the unavailability of data on unplanned admissions post-discharge. While studies have highlighted the significance of 30-day readmission rates as a quality indicator for transsphenoidal pituitary surgery, our study could not evaluate this due to data constraints (24, 25).

## Conclusions

Our results show that MetS does not pose excessive risk of poor postoperative outcomes in terms of in-hospital mortality and unfavorable discharge in patients receiving TSS for pituitary adenoma. However, MetS independently predicted the risk of having PSIs, prolonged hospital stays and higher hospital costs, as well as presence of CSF rhinorrhea. These findings may help clinicians achieve better risk stratification for patients with pituitary adenoma undergoing TSS.

## Data availability statement

The original contributions presented in the study are included in the article/**Supplementary Material**. Further inquiries can be directed to the corresponding author.

## Ethics statement

All data were obtained through request to the Online HCUP Central Distributor. This study conforms to the NIS data-use agreement. Because this study analyzed pre-collected, deidentified data from the NIS and the public were not involved directly, our hospital exempted further Institutional Review Board (IRB) approval after the study protocol was submitted.

## Author contributions

J-LY: Conceptualization, Formal analysis, Investigation, Methodology, Project administration, Validation, Visualization, Writing – original draft. W-CK: Conceptualization, Investigation, Methodology, Validation, Writing – review & editing. Y-HK: Conceptualization, Project administration, Writing – review & editing. M-YC: Conceptualization, Methodology, Project administration, Writing – review & editing. P-YC: Methodology, Writing – review & editing. K-HF: Formal analysis, Investigation, Methodology, Project administration, Validation, Visualization, Writing – original draft.

Funding

The author(s) declare financial support was received for the research, authorship, and/or publication of this article. This work was supported by the Chang Gung Medical Foundation under the Chang Gung Medical Research Project, Grant Number CMRP202200500B0.
