# Supplementary material for: Impact of metabolic syndrome on postoperative outcomes of transsphenoidal pituitary surgery: analysis of U.S. nationwide inpatient sample data 2005–2018
Source: Front Endocrinol (Lausanne). 2024 Mar 25;15:1235441. doi: 10.3389/fendo.2024.1235441 (PMC10999562; doi:10.3389/fendo.2024.1235441)
Supplement: Supplementary file 1 [file Table_1.docx]

| **Supplementary Table S1.**Distribution of the components of MetS in the study cohort. | | | | |
| --- | --- | --- | --- | --- |
| **MetS components** | **Total (n=19,736)** | **MetS** | |  |
|  |  | **Yes (n=2,180)** | **No (n=17,556)** |  |
| BMI ≥ 30 kg/m^2^ | 3306 (16.8) | 1345 (61.7) | 1961 (11.2) |  |
| High blood pressure | 9581 (48.5) | 2101 (96.4) | 7480 (42.6) |  |
| Altered fasting glucose | 3953 (20.1) | 1826 (83.7) | 2127 (12.1) |  |
| Low HDL | 4 (0.0) | 1 (0.0) | 3 (0.0) |  |
| High triglycerides | 4112 (21.0) | 1628 (74.7) | 2484 (14.2) |  |

BMI, body mass index; HDL, high-density lipoprotein; MetS, metabolic syndrome

| **Supplementary Table S2. Associations between study variables and pituitary-related complications, poor outcome, and prolonged LOS.** | | | | | | | | | | | |
| --- | --- | --- | --- | --- | --- | --- | --- | --- | --- | --- | --- |
| **Variables** | **Pituitary-related complications** | | |  | **Poor outcome** | | |  | **Prolonged LOS ^a, b^** | | |
|  | **Univariate** |  | **Multivariate** |  | **Univariate** |  | **Multivariate** |  | **Univariate** |  | **Multivariate** |
|  | **OR (95% CI)** |  | **aOR (95% CI)** |  | **OR (95% CI)** |  | **aOR (95% CI)** |  | **OR (95% CI)** |  | **aOR (95% CI)** |
| **MetS (Yes vs. No)** | 1.05 (0.95, 1.16) |  | 0.97 (0.87, 1.08) |  | **2.09 (1.71, 2.56)** |  | 1.17 (0.93, 1.46) |  | **1.51 (1.36, 1.68)** |  | **1.19 (1.05, 1.34)** |
| **Demography** |  |  |  |  |  |  |  |  |  |  |  |
| Age, years | 1.00 (1.00, 1.00) |  |  |  | **1.05 (1.05, 1.06)** |  | **1.03 (1.02, 1.04)** |  | 1.01 (1.00, 1.01) |  | 1.00 (0.99, 1.00) |
| Sex |  |  |  |  |  |  |  |  |  |  |  |
| Male | **1.20 (1.13, 1.28)** |  | **1.13 (1.06, 1.20)** |  | **1.34 (1.14, 1.58)** |  | 1.17 (0.98, 1.40) |  | 0.97 (0.91, 1.04) |  |  |
| Female | ref |  | ref |  | ref |  | ref |  | ref |  |  |
| Race |  |  |  |  |  |  |  |  |  |  |  |
| White | ref |  | ref |  | ref |  | ref |  | ref |  | ref |
| Black | **1.10 (1.01, 1.20)** |  | 0.95 (0.87, 1.04) |  | **1.91 (1.56, 2.34)** |  | **1.36 (1.08, 1.72)** |  | **2.01 (1.81, 2.24)** |  | **1.46 (1.30, 1.65)** |
| Hispanic | 1.10 (0.98, 1.22) |  | 0.99 (0.89, 1.11) |  | 1.11 (0.85, 1.46) |  | 1.24 (0.92, 1.67) |  | **1.92 (1.69, 2.18)** |  | **1.56 (1.34, 1.81)** |
| Others | **1.20 (1.07, 1.36)** |  | **1.14 (1.01, 1.29)** |  | 1.05 (0.74, 1.47) |  | 1.15 (0.80, 1.65) |  | **1.45 (1.25, 1.68)** |  | **1.38 (1.18, 1.63)** |
| Unknown | 0.89 (0.78, 1.01) |  | 0.88 (0.77, 1.01) |  | **1.30 (1.01, 1.67)** |  | **1.35 (1.03, 1.77)** |  | 1.15 (0.95, 1.40) |  | 1.16 (0.95, 1.43) |
| Household income |  |  |  |  |  |  |  |  |  |  |  |
| Quartile1 | 1.00 (0.91, 1.10) |  |  |  | **1.67 (1.34, 2.07)** |  | **1.31 (1.03, 1.67)** |  | **1.53 (1.37, 1.72)** |  | 1.09 (0.97, 1.24) |
| Quartile2 | 0.99 (0.90, 1.08) |  |  |  | **1.31 (1.03, 1.66)** |  | 1.17 (0.91, 1.50) |  | **1.19 (1.06, 1.34)** |  | 1.00 (0.88, 1.13) |
| Quartile3 | 0.99 (0.91, 1.08) |  |  |  | 1.00 (0.77, 1.28) |  | 1.04 (0.79, 1.35) |  | 1.06 (0.95, 1.19) |  | 1.03 (0.91, 1.15) |
| Quartile4 | ref |  |  |  | ref |  | ref |  | ref |  | ref |
| Primary Payer |  |  |  |  |  |  |  |  |  |  |  |
| Medicare/Medicaid | ref |  | ref |  | ref |  | ref |  | ref |  | ref |
| Private including HMO | **0.87 (0.82, 0.93)** |  | 0.98 (0.92, 1.05) |  | **0.22 (0.18, 0.26)** |  | **0.47 (0.37, 0.59)** |  | **0.56 (0.52, 0.61)** |  | **0.71 (0.64, 0.78)** |
| Self-pay/no-charge/other | 1.07 (0.96, 1.21) |  | 1.02 (0.90, 1.15) |  | **0.32 (0.23, 0.46)** |  | **0.51 (0.35, 0.75)** |  | **1.36 (1.18, 1.56)** |  | 1.16 (0.99, 1.35) |
| Smoking |  |  |  |  |  |  |  |  |  |  |  |
| No | ref |  | ref |  | ref |  | ref |  | ref |  |  |
| Yes | **1.13 (1.05, 1.22)** |  | **1.11 (1.03, 1.20)** |  | **0.77 (0.62, 0.95)** |  | **0.59 (0.47, 0.74)** |  | 0.99 (0.90, 1.08) |  |  |
| CCI |  |  |  |  |  |  |  |  |  |  |  |
| 0 | ref |  | ref |  | ref |  | ref |  | ref |  | ref |
| 1 | **1.31 (1.20, 1.42)** |  | **1.22 (1.12, 1.33)** |  | **3.47 (2.86, 4.22)** |  | **2.60 (2.10, 3.22)** |  | **1.73 (1.57, 1.90)** |  | **1.50 (1.35, 1.67)** |
| 2+ | **1.53 (1.37, 1.72)** |  | **1.34 (1.20, 1.51)** |  | **7.88 (6.42, 9.66)** |  | **3.92 (3.09, 4.97)** |  | **3.14 (2.78, 3.55)** |  | **2.56 (2.24, 2.93)** |
| **Type of pituitary adenoma** | |  |  |  |  |  |  |  |  |  |  |
| Non-secreting | ref |  | ref |  | ref |  | ref |  | ref |  | ref |
| Secreting |  |  |  |  |  |  |  |  |  |  |  |
| Acromegaly | **0.63 (0.56, 0.72)** |  | **0.70 (0.61, 0.79)** |  | **0.23 (0.13, 0.41)** |  | **0.48 (0.26, 0.87)** |  | **0.54 (0.46, 0.63)** |  | **0.78 (0.66, 0.92)** |
| Prolactinoma | 1.15 (0.93, 1.42) |  | 1.24 (0.99, 1.55) |  | 0.68 (0.35, 1.31) |  | 1.19 (0.60, 2.37) |  | **0.69 (0.51, 0.92)** |  | 0.80 (0.59, 1.09) |
| TSHoma | 1.41 (0.42, 4.75) |  | 1.47 (0.41, 5.23) |  | **0.00 (0.00, 0.00)** |  | **0.00 (0.00, 0.00)** |  | 2.67 (0.79, 9.01) |  | **3.61 (1.07, 12.22)** |
| Cushing’s | **0.82 (0.72, 0.94)** |  | 0.93 (0.81, 1.07) |  | 1.07 (0.78, 1.45) |  | **2.45 (1.76, 3.41)** |  | 0.89 (0.77, 1.02) |  | **1.27 (1.08, 1.49)** |
| **Hospital status** |  |  |  |  |  |  |  |  |  |  |  |
| Emergent admission |  |  |  |  |  |  |  |  |  |  |  |
| Yes | **2.70 (2.44, 2.99)** |  | **2.48 (2.23, 2.76)** |  | **4.77 (4.00, 5.69)** |  | **3.82 (3.12, 4.68)** |  | **7.77 (6.91, 8.75)** |  | **6.33 (5.59, 7.17)** |
| No | ref |  | ref |  | ref |  | ref |  | ref |  | ref |
| Weekend admission |  |  |  |  |  |  |  |  |  |  |  |
| No | ref |  | ref |  | ref |  | ref |  | ref |  | ref |
| Yes | **2.14 (1.77, 2.58)** |  | **1.22 (1.01, 1.47)** |  | **2.90 (2.14, 3.91)** |  | 1.19 (0.84, 1.68) |  | **4.47 (3.64, 5.49)** |  | **1.47 (1.20, 1.81)** |
| Hospital bed size |  |  |  |  |  |  |  |  |  |  |  |
| Small | **0.79 (0.65, 0.96)** |  | 0.83 (0.68, 1.01) |  | 0.71 (0.46, 1.09) |  | 0.93 (0.61, 1.41) |  | **0.60 (0.48, 0.75)** |  | **0.75 (0.62, 0.90)** |
| Medium | 0.90 (0.80, 1.01) |  | **0.86 (0.77, 0.97)** |  | **1.35 (1.07, 1.69)** |  | **1.31 (1.04, 1.66)** |  | 1.11 (0.97, 1.27) |  | 1.07 (0.94, 1.22) |
| Large | ref |  | ref |  | ref |  | ref |  | ref |  | ref |
| Hospital location/teaching status | |  |  |  |  |  |  |  |  |  |  |
| Rural | **0.62 (0.53, 0.74)** |  | **0.63 (0.53, 0.75)** |  | 0.71 (0.35, 1.42) |  |  |  | 1.04 (0.87, 1.25) |  |  |
| Urban nonteaching | **0.82 (0.72, 0.92)** |  | **0.77 (0.68, 0.87)** |  | 0.99 (0.77, 1.28) |  |  |  | 1.01 (0.86, 1.18) |  |  |
| Urban teaching | ref |  | ref |  | ref |  | ref |  | ref |  |  |
| Hospital region |  |  |  |  |  |  |  |  |  |  |  |
| Northeast | ref |  |  |  | ref |  | ref |  | ref |  | ref |
| Midwest | **1.19 (1.04, 1.37)** |  |  |  | 0.89 (0.70, 1.15) |  | 0.83 (0.63, 1.09) |  | 0.94 (0.77, 1.14) |  | 1.01 (0.83, 1.22) |
| South | 1.06 (0.93, 1.20) |  |  |  | **0.70 (0.56, 0.88)** |  | **0.59 (0.46, 0.75)** |  | 1.16 (0.98, 1.37) |  | 1.03 (0.87, 1.21) |
| West | 1.12 (0.97, 1.30) |  |  |  | **0.50 (0.38, 0.66)** |  | **0.51 (0.38, 0.67)** |  | **0.79 (0.66, 0.95)** |  | **0.77 (0.64, 0.93)** |

^a^ Excluded In-hospital mortality patients.

^b^ LOS >75^th^ percentile (5 days).

LOS, length of stay; HMO, Health Maintenance Organization; CML; CCI, Charlson Comorbidity Index; TSHoma, thyroid-stimulating hormone (TSH)-secreting pituitary adenoma; MetS, metabolic syndrome.

Significant values are shown in bold.

NA: (not applicable) no event occurred in the group.

| **Supplementary Table S3. Associations between study variables, PSI and total hospital costs.** | | | | | | | |
| --- | --- | --- | --- | --- | --- | --- | --- |
| **Variable** | **PSI** | | |  | **Total hospital cost (per 1000 dollars)** | | |
|  | **Univariate** |  | **Multivariate** |  | **Univariate** |  | **Multivariate** |
|  | **OR (95% CI)** |  | **aOR (95% CI)** |  | **β** **(95% CI)** |  | **a β** **(95% CI)** |
| **Metabolic Syndrome (Yes vs No)** | **1.77 (1.46, 2.14)** |  | **1.31 (1.07, 1.59)** |  | **15.29 (11.62, 18.96)** |  | **8.63 (4.98, 12.29)** |
| **Demography** |  |  |  |  |  |  |  |
| Age, years | **1.02 (1.02, 1.03)** |  | 1.01 (1.00, 1.02) |  | **0.24 (0.16, 0.32)** |  | 0.01 (-0.08, 0.11) |
| Sex |  |  |  |  |  |  |  |
| Male | **1.28 (1.11, 1.47)** |  | 1.13 (0.97, 1.31) |  | **5.16 (3.03, 7.29)** |  | **3.08 (1.01, 5.15)** |
| Female | ref |  | ref |  | ref |  | ref |
| Race |  |  |  |  |  |  |  |
| White | ref |  |  |  | ref |  | ref |
| Black | 1.22 (0.99, 1.49) |  |  |  | **11.06 (7.18, 14.95)** |  | **7.83 (4.08, 11.57)** |
| Hispanic | 1.19 (0.93, 1.51) |  |  |  | **21.02 (15.60, 26.44)** |  | **15.84 (10.84, 20.84)** |
| Others | 1.22 (0.94, 1.58) |  |  |  | **10.25 (5.15, 15.34)** |  | **5.48 (0.53, 10.42)** |
| Unknown | 1.16 (0.92, 1.45) |  |  |  | **-18.40 (-22.77, -14.02)** |  | **-17.55 (-21.90, -13.21)** |
| Household income |  |  |  |  |  |  |  |
| Quartile1 | **1.25 (1.03, 1.53)** |  |  |  | 1.95 (-1.72, 5.61) |  |  |
| Quartile2 | 1.09 (0.90, 1.33) |  |  |  | -0.67 (-4.74, 3.40) |  |  |
| Quartile3 | 1.09 (0.90, 1.33) |  |  |  | -2.37 (-5.59, 0.86) |  |  |
| Quartile4 | ref |  |  |  | ref |  |  |
| Primary Payer |  |  |  |  |  |  |  |
| Medicare/Medicaid | ref |  | ref |  | ref |  | ref |
| Private including HMO | **0.50 (0.43, 0.58)** |  | **0.77 (0.65, 0.91)** |  | **-16.74 (-19.54, -13.94)** |  | **-8.41 (-11.24, -5.57)** |
| Self-pay/no-charge/other | **0.70 (0.54, 0.91)** |  | 0.91 (0.69, 1.21) |  | -4.01 (-9.67, 1.66) |  | -2.76 (-8.56, 3.03) |
| Smoking |  |  |  |  |  |  |  |
| No | ref |  |  |  | ref |  |  |
| Yes | 0.90 (0.76, 1.07) |  |  |  | -0.20 (-2.85, 2.45) |  |  |
| CCI |  |  |  |  |  |  |  |
| 0 | ref |  | ref |  | ref |  | ref |
| 1 | **2.44 (2.05, 2.89)** |  | **2.11 (1.77, 2.52)** |  | **18.47 (14.34, 22.61)** |  | **13.98 (10.03, 17.92)** |
| 2+ | **4.70 (3.88, 5.70)** |  | **3.39 (2.74, 4.19)** |  | **43.29 (35.54, 51.04)** |  | **34.39 (26.77, 42.02)** |
| **Type of pituitary adenoma** | |  |  |  |  |  |  |
| Non-secreting | ref |  |  |  | ref |  | ref |
| Secreting |  |  |  |  |  |  |  |
| Acromegaly | **0.70 (0.52, 0.95)** |  |  |  | **-7.07 (-10.74, -3.39)** |  | -1.66 (-5.00, 1.68) |
| Prolactinoma | 0.91 (0.55, 1.51) |  |  |  | 0.44 (-6.91, 7.80) |  | 4.64 (-1.97, 11.25) |
| TSHoma | 2.52 (0.32, 19.61) |  |  |  | -9.71 (-23.78, 4.35) |  | -8.93 (-27.73, 9.86) |
| Cushing’s | 0.95 (0.73, 1.25) |  |  |  | -1.26 (-6.18, 3.66) |  | 3.90 (-0.99, 8.78) |
| **Hospital status** |  |  |  |  |  |  |  |
| Emergent admission |  |  |  |  |  |  |  |
| Yes | **2.50 (2.12, 2.94)** |  | **1.96 (1.64, 2.34)** |  | **47.36 (41.13, 53.59)** |  | **40.41 (34.24, 46.58)** |
| No | ref |  | ref |  | ref |  | ref |
| Weekend admission |  |  |  |  |  |  |  |
| No | ref |  | ref |  | ref |  | ref |
| Yes | **2.31 (1.74, 3.05)** |  | 1.36 (0.99, 1.88) |  | **30.90 (23.15, 38.65)** |  | 4.12 (-3.85, 12.10) |
| Hospital bed size |  |  |  |  |  |  |  |
| Small | 0.91 (0.68, 1.21) |  |  |  | **-13.02 (-20.01, -6.04)** |  | **-10.93 (-17.94, -3.92)** |
| Medium | 1.19 (0.97, 1.47) |  |  |  | **-4.72 (-9.32, -0.12)** |  | **-4.40 (-8.58, -0.22)** |
| Large | ref |  |  |  | ref |  | ref |
| Hospital location/teaching status | |  |  |  |  |  |  |
| Rural | 0.65 (0.35, 1.22) |  |  |  | **-22.85 (-33.74, -11.97)** |  | **-20.27 (-33.02, -7.52)** |
| Urban nonteaching | 0.95 (0.76, 1.19) |  |  |  | -1.94 (-6.35, 2.46) |  | -3.05 (-7.27, 1.18) |
| Urban teaching | ref |  |  |  | ref |  | ref |
| Hospital region |  |  |  |  |  |  |  |
| Northeast | ref |  |  |  | ref |  | ref |
| Midwest | 1.16 (0.90, 1.50) |  |  |  | **-13.88 (-22.58, -5.17)** |  | -6.93 (-15.69, 1.84) |
| South | 1.04 (0.83, 1.32) |  |  |  | **-16.01 (-24.54, -7.48)** |  | **-16.82 (-25.08, -8.56)** |
| West | 1.07 (0.83, 1.38) |  |  |  | 1.40 (-8.26, 11.07) |  | 3.43 (-5.72, 12.58) |

PSI, patient safety indicator; HMO, Health Maintenance Organization; CML; CCI, Charlson Comorbidity Index; TSHoma, thyroid-stimulating hormone (TSH)-secreting pituitary adenoma.

Significant values are shown in bold.

| **Supplementary Table S4. Associations between ≥ 2 MetS criteria and the outcomes.** | | | | |
| --- | --- | --- | --- | --- |
| **Outcomes** | **≥ 2 MetS criteria**  **(n=6,078 vs. 12,998)** | **Univariate** |  | **Multivariate** |
|  |  | **Crude OR (95% CI) / β (95% CI)** |  | **Adjusted OR (95% CI) / β (95% CI)** |
| **Pituitary-related complications ^a^** | Yes vs. No | 1.01 (0.94, 1.08) |  | 0.94 (0.88, 1.01) |
| **Poor outcomes ^b^** | Yes vs. No | **1.73 (1.47, 2.04)** |  | 0.91 (0.75, 1.09) |
| In-hospital mortality ^c^ | Yes vs. No | 1.43 (0.88, 2.32) |  | 0.80 (0.46, 1.39) |
| Unfavorable discharge ^d, h^ | Yes vs. No | **1.77 (1.48, 2.10)** |  | 0.91 (0.75, 1.10) |
| **Prolonged LOS ^e, h, i^** | Yes vs. No | **1.41 (1.30, 1.52)** |  | **1.17 (1.07, 1.28)** |
| **PSI ^f^** | Yes vs. No | **1.58 (1.37, 1.83)** |  | **1.17 (1.01, 1.37)** |
| **Total hospital cost (per 1000 dollars) ^g, j^** | Yes vs. No | **10.59 (8.16, 13.02)** |  | **5.37 (2.76, 7.99)** |
| Variables with p<0.05 in univariate analysis were adjusted for multivariate analysis. | | | | |
| Significant values are shown in bold. | | | | |
| In multivariate analysis, models were adjusted using the following covariates:  ^a^ sex, race, primary payer, categorical CCI, type of pituitary adenoma, emergent admission, weekend admission, hospital bed size, and hospital location/teaching status.  ^b^ age, sex, race, household income, primary payer, smoking, categorical CCI, type of pituitary adenoma, emergent admission, weekend admission, hospital bed size, and hospital region.  ^c^ age, sex, race, primary payer, categorical CCI, type of pituitary adenoma, emergent admission, weekend admission, hospital bed size, and hospital location/teaching status.  ^d^ age, sex, race, household income, primary payer, categorical CCI, type of pituitary adenoma, emergent admission, weekend admission, hospital bed size, and hospital region.  ^e^ age, race, household income, primary payer, categorical CCI, type of pituitary adenoma, emergent admission, weekend admission, hospital bed size, and hospital region.  ^f^ age, sex, primary payer, categorical CCI, emergent admission, and weekend admission.  ^g^ age, sex, race, primary payer, categorical CCI, type of pituitary adenoma, emergent admission, weekend admission, hospital bed size, hospital location/teaching status, and hospital region. | | | | |
| ^h^ Excluded in-hospital mortality patients. | | | | |
| ^i^ LOS >75^th^ percentile (5 days) | | | | |
| ^j^ Adjusted β was used to total hospital cost (per 1000 dollars). | | | | |
| Abbreviations: LOS, length of stay; CCI, Charlson comorbidity index; OR, odds ratio; CI, confidence interval; MetS, metabolic syndrome; PSI, patient safety indicator. | | | | |
| **Supplementary Table S5. Associations between ≥ 2 MetS criteria and specific pituitary-related complications.** | | | | |
| **Pituitary-related complication** | **≥ 2 MetS criteria**  **(n=6,078 vs. 12,998)** | **Univariate** |  | **Multivariate** |
|  |  | **Crude OR (95% CI)** |  | **Adjusted OR (95% CI)** |
| Pituitary apoplexy ^a^ | Yes vs. No | 0.97 (0.80, 1.18) |  | 1.05 (0.84, 1.30) |
| Panhypopituitarism ^b^ | Yes vs. No | 1.10 (0.99, 1.23) |  | 0.96 (0.85, 1.08) |
| Corticoadrenal insufficiency ^c^ | Yes vs. No | 1.05 (0.93, 1.19) |  | 0.97 (0.85, 1.10) |
| Acquired hydrocephalus ^d^ | Yes vs. No | 1.00 (0.72, 1.40) |  | 0.75 (0.52, 1.08) |
| Meningitis ^e^ | Yes vs. No | 1.07 (0.54, 2.10) |  | 0.88 (0.42, 1.82) |
| CSF rhinorrhea ^f^ | Yes vs. No | 1.14 (0.998, 1.31) |  | 1.15 (0.998, 1.32) |
| Diabetes insipidus ^g^ | Yes vs. No | **0.72 (0.65, 0.79)** |  | **0.85 (0.77, 0.95)** |
| Hypernatremia ^h^ | Yes vs. No | **1.32 (1.15, 1.50)** |  | 1.05 (0.91, 1.21) |
| SIADH ^i^ | Yes vs. No | 0.84 (0.69, 1.03) |  | **0.77 (0.61, 0.96)** |
| Variables with p<0.05 in univariate analysis were adjusted for multivariate analysis. | | | | |
| Significant values are shown in bold. | | | | |
| In multivariate analysis, models were adjusted with the following covariates:  ^a^ age, race, categorical CCI, type of pituitary adenoma, emergent admission, and weekend admission.  ^b^ age, sex, primary payer, smoking, categorical CCI, type of pituitary adenoma, emergent admission, weekend admission, hospital bed size, hospital location/teaching status, and hospital region.  ^c^ sex, primary payer, smoking, categorical CCI, type of pituitary adenoma, emergent admission, weekend admission, hospital bed size, hospital location/teaching status, and hospital region.  ^d^ age, sex, primary payer, categorical CCI, type of pituitary adenoma, emergent admission, and weekend admission.  ^e^ categorical CCI, type of pituitary adenoma, emergent admission, weekend admission, and hospital location/teaching status.  ^f^ sex, race, emergent admission, hospital bed size, and hospital location/teaching status.  ^g^ age, sex, primary payer, categorical CCI, type of pituitary adenoma, emergent admission, weekend admission, and hospital location/teaching status.  ^h^ age, sex, primary payer, categorical CCI, type of pituitary adenoma, emergent admission, and weekend admission.  ^i^ age, sex, race, household income, categorical CCI, type of pituitary adenoma, emergent admission, weekend admission, and hospital location/teaching status. | | | | |
| Abbreviations: CSF, cerebrospinal fluid; SIADH, syndrome of inappropriate antidiuretic hormone secretion; CCI, Charlson comorbidity index; OR, odds ratio; CI, confidence interval; MetS, metabolic syndrome. | | | | |
